# Visual explanations for polyp detection: How medical doctors assess intrinsic versus extrinsic explanations

**DOI:** 10.1371/journal.pone.0304069

**Published:** 2024-05-31

**Authors:** Steven Hicks, Andrea Storås, Michael A. Riegler, Cise Midoglu, Malek Hammou, Thomas de Lange, Sravanthi Parasa, Pål Halvorsen, Inga Strümke

**Affiliations:** 1 Department of Holistic Systems, SimulaMet, Oslo, Norway; 2 Department of Computer Science, Norwegian University of Science and Technology, Trondheim, Norway; 3 Medical Department, Sahlgrenska University Hospital-Mölndal, Region Västra Götaland, Mölndal, Sweden; 4 Department of Gastroenterology, Swedish Medical Group, Seattle, WA, United States of America; 5 Department of Computer Science, Oslo Metropolitan University, Oslo, Norway; 6 Department of Computer Science, UIT The Arctic University of Norway, Tromsø, Norway; 7 Department of Molecular and Clinical Medicine, Sahlgrenska Academy, University of Gothenburg, Gothenburg, Sweden; University of Pisa, ITALY

## Abstract

Deep learning has achieved immense success in computer vision and has the potential to help physicians analyze visual content for disease and other abnormalities. However, the current state of deep learning is very much a black box, making medical professionals skeptical about integrating these methods into clinical practice. Several methods have been proposed to shed some light on these black boxes, but there is no consensus on the opinion of medical doctors that will consume these explanations. This paper presents a study asking medical professionals about their opinion of current state-of-the-art explainable artificial intelligence methods when applied to a gastrointestinal disease detection use case. We compare two different categories of explanation methods, intrinsic and extrinsic, and gauge their opinion of the current value of these explanations. The results indicate that intrinsic explanations are preferred and that physicians see value in the explanations. Based on the feedback collected in our study, future explanations of medical deep neural networks can be tailored to the needs and expectations of doctors. Hopefully, this will contribute to solving the issue of black box medical systems and lead to successful implementation of this powerful technology in the clinic.

## Introduction

Deep learning is becoming an increasingly popular method for analyzing medical image data to perform tasks such as the detection of lesions or classification of diseases. However, despite the prevalent use of deep learning in medical research, deep learning is rarely implemented in a clinical setting [1]. Several factors make the use of deep learning-based systems in medicine problematic, such as the potential legal implications of incorrect diagnoses or the presence of unintended biases against a specific race or gender. Many of these issues stem from a general lack of explainability and interpretability in applied deep learning algorithms. Deep neural networks are complex statistical models consisting of millions, if not billions, of parameters, making it difficult to understand what reasoning lies behind a specific prediction. Explainable artificial intelligence (XAI) aims to solve the question of explainability and interpretability by providing methods that aim to explain the internal decision process of the neural network in a more digestible and understandable way. Several XAI methods have been proposed, where SHapley Additive exPlanations (SHAP) [2] and salient-based explanations such as Gradient-weighted Class Activation Mapping (GradCAM) [3] are among the most popular techniques for image-based models. These methods provide an overlay that signifies what regions of an image contributed to the predicted output, making them relatively easy to understand among a non-technical audience. Several studies stress the importance of explanations that can be interrupted by non-tech-savvy users such as physicians or clinicians to better understand the underlying reasoning behind a prediction [4]. However, there is no consensus on which explanation methods are preferred or whether medical professionals actually find them useful. Similar studies have been conducted in the general population [5], however, a study on the opinion of domain experts on XAI in the field of gastrointestinal (GI) has not been conducted to our knowledge.

XAI is the sub-field of artificial intelligence (AI) dedicated to explaining AI systems that are opaque or non-intuitive to humans. Different end-users have different needs for explanations, ranging from the developer who wishes to improve the system and ensure its robustness to the doctor using the system as decision support in the clinic and wanting to verify the veracity of the system’s findings and potentially communicate this to the patient. Recent advances in the XAI literature almost exclusively refer to methods designed to explain the behavior of complex machine learning (ML) models. The XAI literature is large and rapidly developing, and we do not attempt to give an overview here. For simplicity, we compare two categories of XAI methods, intrinsic and extrinsic explanations. Intrinsic explanations cover the methods that aim to explain a model’s internals through analyzing the model weights, mostly including saliency-based methods. Extrinsic explanations aim to explain the model using external input such as SHAP [2] or Local Interpretable Model-Agnostic Explanations (LIME) [6].

**Fig 1:**
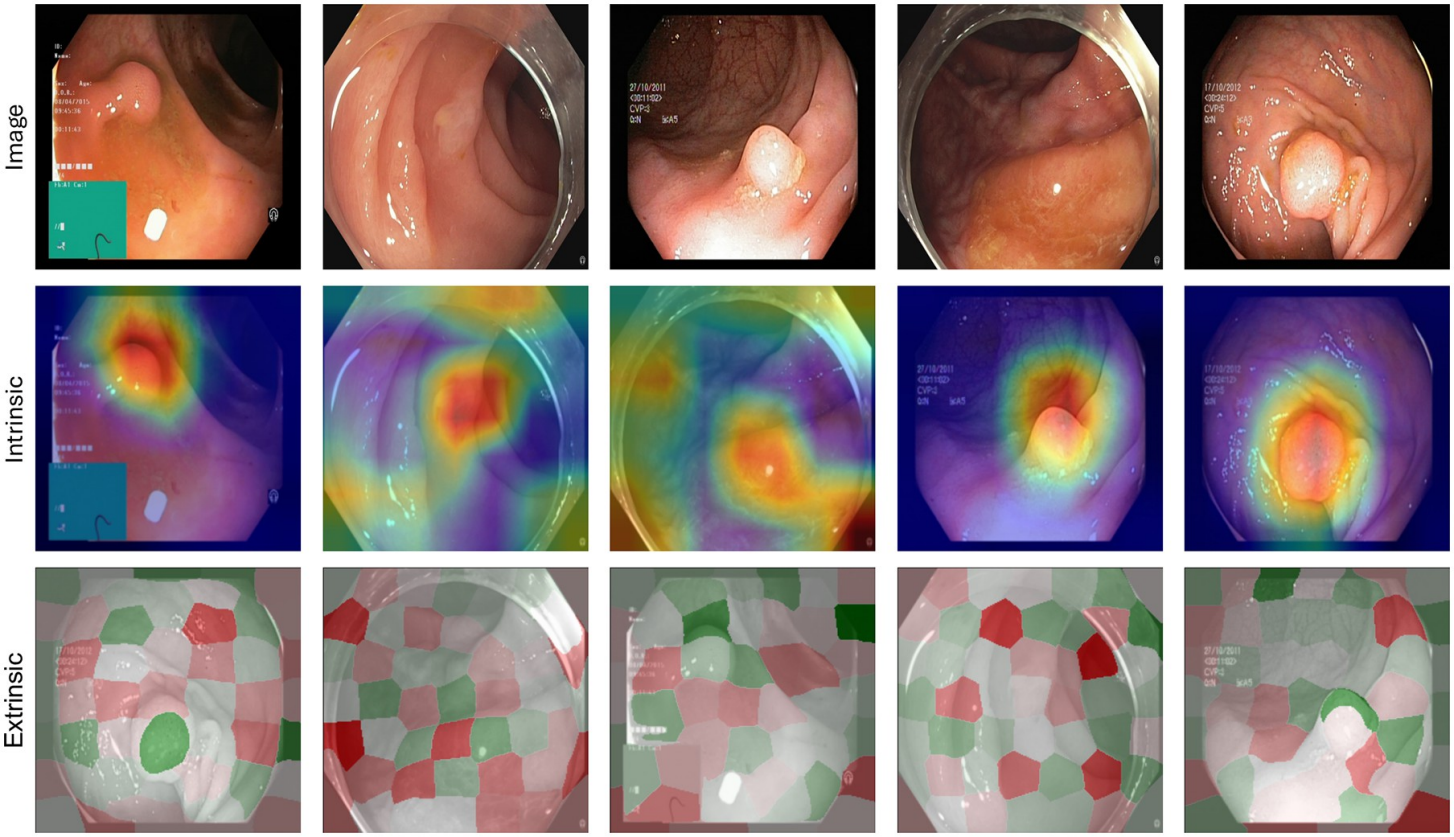
Five cases taken from the survey presented to each participant. The top row shows the image that was passed through the model to generate a prediction. The second row shows the intrinsic explanation method (GradCAM) used to explain the prediction. The last row shows the extrinsic explanation method used to explain the prediction (SHAP).

As previously explained, intrinsic explanations aim to explain the predictions of a model by looking at the internal weights to provide some reasoning behind a specific output and are the most common method for explaining deep neural networks. There is a large variety of such methods available, including [3, 7–13], and it is not obvious which, if any, method is superior. In this study, we use GradCAM, which is arguably the most popular method for intrinsic-based visual explanations. Moreover, GradCAM has passed several sanity checks, as opposed to other popular intrinsic explanation methods [14]. GradCAM highlights the important parts of the image for a predicted class based on activated neurons in a specific layer of a neural network model. First, the gradients for the class are computed with respect to the feature maps of a layer in the model. Weights that are important for predicting a selected class are then obtained in order to identify which parts of the image contribute to the prediction, which can then be mapped back to the input. The heat maps follow the standard Jet color mapping, which consists of a gradient from red to green and green to blue, where red indicates the most important areas of the image, yellow indicates less important areas, and blue marks the least important areas. Examples from the study can be seen in row 2 of Fig 1. The user selects which layer of the model to extract the heat maps from. Usually, later convolutional layers, i.e., layers that are close to the output layer, are helpful in highlighting higher-level details. Moreover, the heat maps will depend on the model architecture since this will affect the activations for the selected layer. Consequently, many different heat maps can be generated for the same image. This means that some heat maps might be regarded useful by users, while others might not. An advantage of GradCAM is that it visually explains the inner workings of the neural network model, making it easier to understand.

Extrinsic explanation methods, which also fall into categories referred to as model-agnostic and model-independent, treat the model as a phenomenon and present information about its emergent behavior. In the case of image classification, the presentation is visually similar to the aforementioned class of methods, that is, a heat map superimposed on the image, but involves occlusion [15] and perturbation of image segments, for which there are significantly fewer methods of this kind available. The SHAP [2] library is widely used for explaining ML models, popular for its solid theoretical foundation in game theory. The name of the SHAP package [2] is an acronym for Shapley additive explanations, and the method is based on the Shapley decomposition, which is a solution concept from cooperative game theory. SHAP simulates feature absence by sampling from a background data set. Therefore, the resulting SHAP value indicates how much the value of a feature causes the model’s prediction to move away from the average prediction across the data. For images, systematic removal of all pixels from which an image consists is infeasible, so SHAP instead groups pixels based on their relative characteristics. For this, SHAP uses an external computer vision-based segmentation algorithm [16]. The coarseness of the final SHAP heat map is adjusted by the user, and smaller pixel groups require more computational resources. In summary, SHAP, when applied to images, produces a heat map indicating which parts of the image support and oppose a classification relative to a chosen background data set. The built-in color scheme, also used in our study, uses shades of pink and blue superimposed on the image to indicate that a region supports (pink) or opposes (blue) the model prediction. Examples of the study can be seen in row 3 of Fig 1.

This article presents a study on collecting feedback from medical professionals on the current state-of-the-art XAI methods used to explain the prediction of deep learning models based on computer vision. The study was carried out using the automatic detection of colon polyps as a use case, where a deep learning-based model is tasked with classifying images as either containing a polyp or not. Polyps are lesions within the bowel detectable as mucosal outgrows. Polyps are flat, elevated, or pedunculated and distinguished from normal mucosa by color and surface pattern. Most intestinal polyps are harmless, but some have the potential to become cancer. Therefore, the detection and removal of polyps are essential to prevent the development of colorectal cancer. Since medical doctors often overlook polyps, automatic detection would most likely improve examination quality. Furthermore, automatic computer-aided polyp detection would be valuable for diagnosis, assessment, and reporting, and is currently a very popular research area in medical AI [17, 18]. Due to its timeliness and clear objective, we find that the GI use case is a perfect case study to evaluate XAI methods for medical use cases. Note that this study only looks at the explanation of two-dimensional visual prediction models. Rather than developing another XAI method, the main contributions of our work are to identify what medical domain experts need from model explanations and evaluate whether existing XAI methods meet those needs. Leveraging this information in future AI systems can facilitate the implementation of the systems in clinical practice.

## Materials and methods

The primary objective of this study was to quantify the value of current state-of-the-art XAI methods from the perspective of medical doctors. During the course of five months (September 2021—February 2022), we sent a survey invitation to a number of different medical doctors located in different parts of the world together with a short video explaining the study (https://youtu.be/JJ8uc5gReko). In this section, we describe the motivation and thought process behind the building of the study. This includes the development of the online survey, the implementation of the deep learning model used to generate the question cases, and the dataset used.

### Ethics statement

No compensation was given for participating in the study. All participants were confirmed to be over the age of 18 years and each participant provided their written consent through a checkbox. Before the study started, it was reviewed and approved by the Norwegian Regional Committees for Medical and Health Research Ethics (#203229). All responses to the survey were anonymized.

### Survey

The survey was built using the open-source framework *Huldra*, which is a framework to collect crowd-sourced feedback on multimedia assets (https://github.com/simula/huldra). The framework allows for the collection of participant responses in a storage bucket hosted on the cloud, from where they can be retrieved in real-time by survey organizers, using credentials, immediately after the first interaction of each participant. The survey consisted of four distinct parts; registration, orientation, case questionnaire, and feedback.

The first part of the survey asked participants to register with their name (optional), email (optional), country, academic degree(s), the field of expertise, and how many years they have been active in the field. The second part orientated the participants on what they could expect from the survey and provided some background information on the two explanation methods that would be compared during the survey. The main part of the survey consisted of 10 cases in which a model predicted whether an image of the GI tract contained a polyp or not. The cases were selected based on three scenarios; high-confidence correct polyp prediction (7 images), not confident correct polyp prediction (1 image), and high confident incorrect polyp prediction (2 images). To avoid bias about which order the examples were shown, the images were randomly shuffled on participant-level, meaning that the order differed between the participants. The prediction was shown together with the image, along with the two explanation methods that support the prediction. Here, participants were asked to select which of the two explanation methods they found the most helpful. The cases were shuffled on a per-participant basis, meaning that the order in which the cases were shown was not the same between participants. As the attention span may differ between the first and last cases, we wanted to avoid any bias that could be introduced through the ordering of the different cases. The last part of the survey contained a feedback form consisting of 14 questions (see Table 1) that were intended to derive a summary of the doctor’s general perception of the two explanation methods. The participants also received a summary of their previous responses, where they had the option to go back and review/change the selection for specific cases.

**Table 1:** The questions that were asked to the participants in the final feedback form. Please note that *Explanation A* refers to the intrinsic explanation and *Explanation B* is the extrinsic explanation.

Type	Question
Likert scale (1–10)	Explanation (A) increased my understanding of the result.
	Explanation (B) increased my understanding of the result.
	Explanation (A) increased my trust in the AI model.
	Explanation (B) increased my trust in the AI model.
	I found the colors used to visualize explanation (A) to be appropriate.
	I found the colors used to visualize explanation (B) to be appropriate.
	Explanation (A) frequently highlighted the correct area in the image.
	Explanation (B) frequently highlighted the correct area in the image.
	It is important that an explanation accompanies a prediction.
Multiple choice	Do you prefer to have an explanation, or would you rather only know the prediction?
	Which type of explanation would be useful in clinical practice?
	Would you prefer that explanations for the predictions be shown during or after the procedure?
Free form	What do you think of explanation method (A)?
	What do you think of explanation method (B)?

### Dataset

The dataset used to sample the case images and train the deep neural network was *Kvasir* [19], which is an open GI dataset consisting of different findings from the GI tract. *Kvasir* consists of images annotated and verified by medical doctors (experienced endoscopists), including several classes showing anatomical landmarks, pathological findings, or endoscopic procedures in the GI tract, with hundreds of images for each class. Anatomical landmarks include the Z-line, pylorus, cecum, etc., while the pathological finding includes esophagitis, polyps, ulcerative colitis, etc. In addition, several sets of images related to the removal of lesions are also provided, like *dyed and lifted polyp* and *dyed resection margins*. The dataset contains images with different resolutions from 720 × 576 up to 1920 × 1072 pixels. Some of the included classes of images have a green box in the lower-left corner that illustrates the position and configuration of the endoscope inside the bowel using an electromagnetic imaging system (ScopeGuide, Olympus Europe). Examples from the dataset can be seen in row 1 of Fig 1.

### Implementation of explanation methods

The model used to classify the images and generate the explanations was a machine learning (CNN) based on the ResNet [20] architecture implemented in PyTorch and trained on a modified version of the aforementioned Kvasir [19] dataset. The dataset was modified to accommodate the use case of distinguishing between images containing polyps and images of a clean colon. Regarding the explanation methods, we used Captum [21] provided by PyTorch for the extrinsic explanations and an open implementation of GradCAM (https://github.com/vickyliin/gradcamç_plus_plus-pytorch) for the intrinsic explanations. The model was trained on what can be considered consumer-grade hardware, containing a Nvidia RTX 3090 GPU and an Intel i9 processor. The source code and more details on the implementation of the model used to generate the explanations can be found in our GitHub repository: https://github.com/simula/xai-gi-survey.

## Results

**Fig 2:**
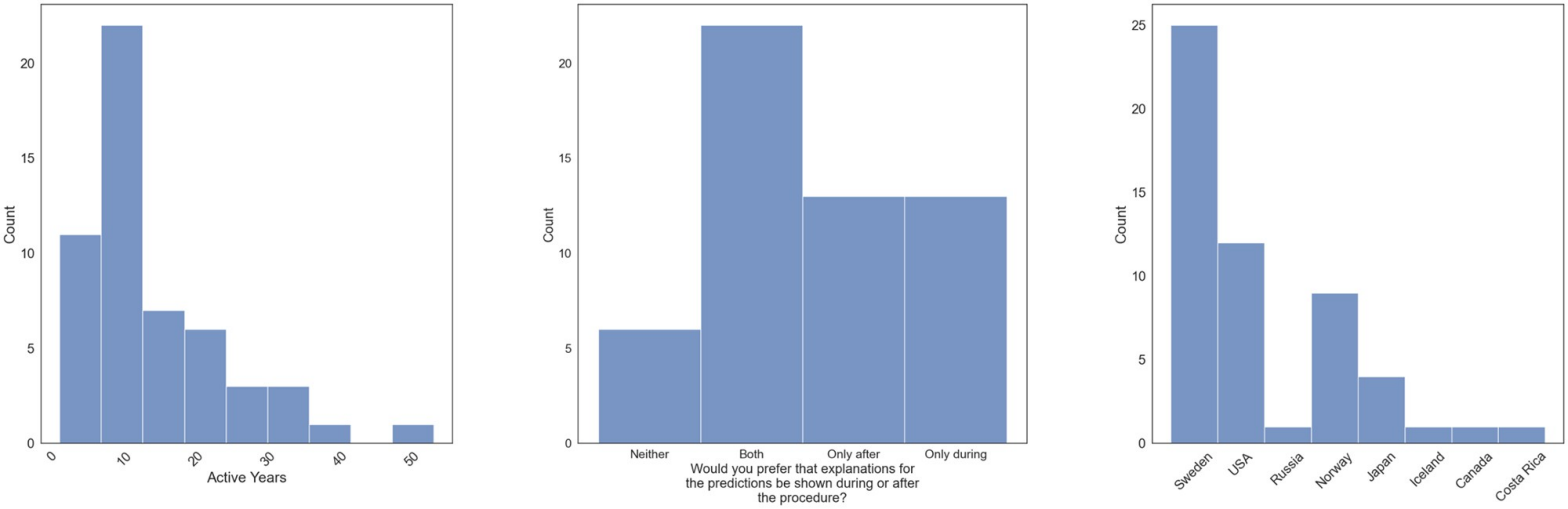
Plots presenting some statistics about the participants included in the study. (a) Number of active years in the medical field. (b) The degree(s) obtained by the participants. (c) The country that the participant comes from.

The survey collected a total of 57 responses. Of these, 54 were used in the final analysis. Among the initial responses were a few non-medical workers, including AI specialists and marketers. As the primary motivation behind this study was to better understand the opinion of medical professionals working with AI, we decided to filter out these and only keep the responses of the participants working in the medical field. Apart from non-medical participants, we also filtered out any incomplete submissions. In the end, the remaining participants came from eight different countries with varying amounts of experience in the medical field, ranging from just a few years to over 50 years. Fig 2 shows some plots regarding the participants’ statistics in terms of active years in the field, obtained degree(s), and the country from which the participants come from. The rest of this section is organized by question category, where we present a summary of the participants’ responses for the explanation case questions, Likert questions, multiple choice questions, and free-form questions.

### Explanation case responses

To ensure a robust analysis of the different perspectives represented by the participants, we used the Statistical Package for the Social Sciences (SPSS) to perform an intra-reliability test, a statistical measure that assesses the degree of agreement between the study participants for all explanation case responses (see Table 1) [22]. An intraclass correlation coefficient (ICC) value of 1 corresponds to perfect agreement. The values between 0.70 and 0.79 are considered fair, the values between 0.80 and 0.89 are good, while the values of 0.90 and above are excellent with respect to clinical relevance [23]. One of the strengths of ICC is that it takes the magnitude of disagreement between the raters; larger disagreements lead to lower ICC values than smaller disagreements [23].

**Fig 3:**
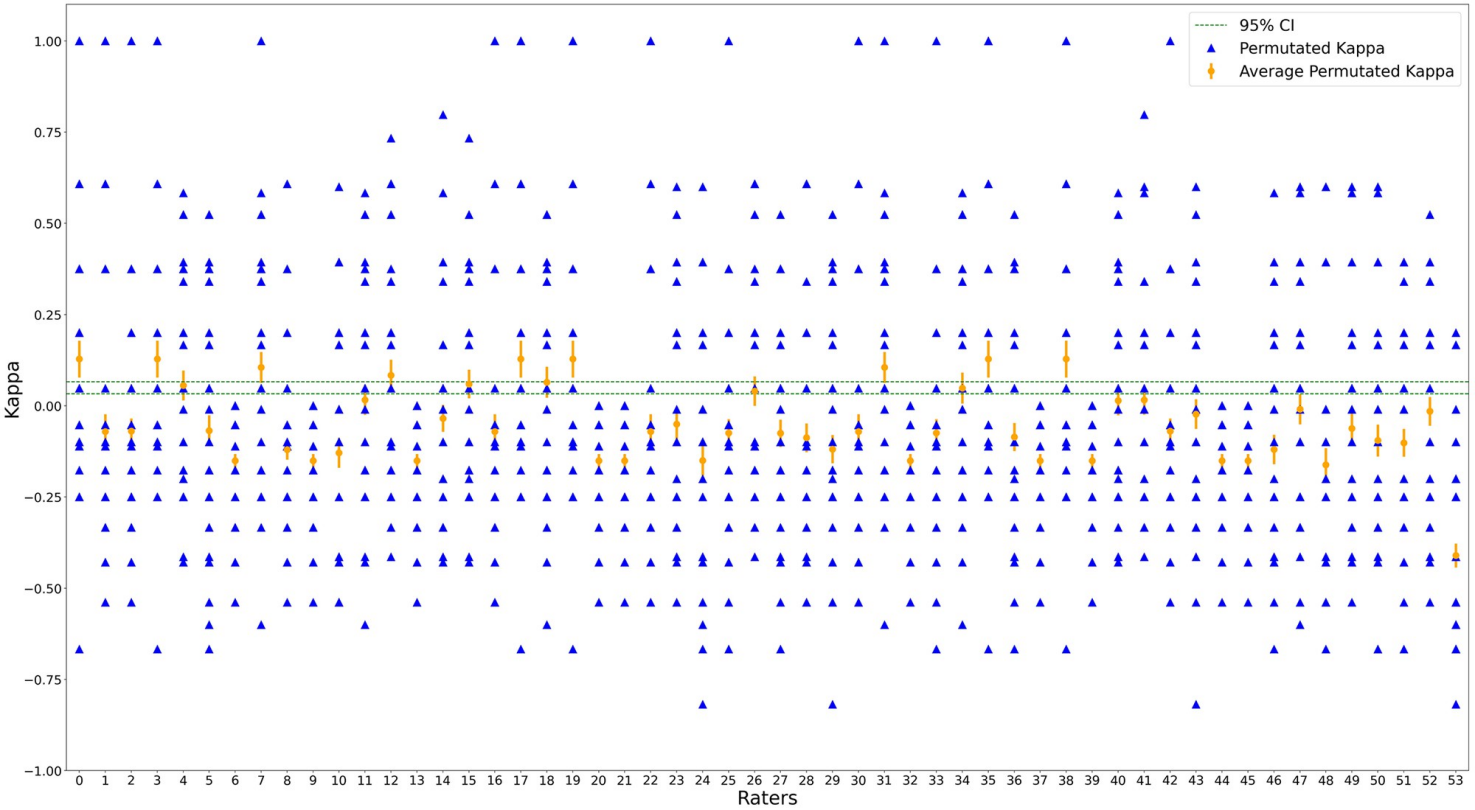
The distribution of premuted kappa values across rater combinations. Blue triangles indicate individual kappa values for each rater pair. The mean kappa value and its 95% CI are depicted by solid orange lines. The green dashed lines represent the global lower and upper bounds of the 95% CI for the Fleiss kappa. (a) Method A increased trust. (b) Method B increased trust. (c) Method A increased understanding. (d) Method B increased understanding. (e) Method A was spatially correct. (f) Method B was spatially correct. (g) Method A used appropriate colors. (h) Method B used appropriate colors. (i) Explanations are important.

The ICC was calculated for all the explanation cases to assess the level of agreement between the responses from the study participants. From Table 2, we see that the average measure of the ICC is 0.794 with a range of 0.559 to 0.938, which means that the agreement is fair. Moving from the ICC to a more nuanced measure of agreement, we used the Fleiss’ kappa statistic to examine the inter-rater reliability within our study. The results, as detailed in Table 3, show a Fleiss’ kappa value of 0.049. Unlike the ICC, The Fleiss’ kappa value signals poor agreement among the study participants. This discrepancy underscores the complexity of measuring agreement and highlights the necessity of using multiple statistical measurements to capture the full spectrum of inter-rater dynamics. Fig 3 displays a plot of kappa values [24], providing a visual representation of inter-rater reliability assessments. Each point represents the kappa statistic for a specific rater combination, illustrating the degree of agreement among the raters.

**Table 2:** Single and average ICC calculated for all explanation cases to measure the agreement between the answers of the participants.

	Interclass Correlation	95% Confidence Interval		F Test with True Value 0			
		Lower Bound	Upper Bound	Value	df1	df2	Sig
Single Measures	0.067	0.023	0.220	4.852	9	477	< 0.000
Average Measures	0.794	0.559	0.938	4.852	9	477	< 0.000

**Table 3:** The Fleiss kappa value measuring the reliability of agreement between the answers of the participants.

	Kappa	Asymptotic			Asymptotic 95% Confidence Interval	
		Standard Error	z	Sig	Lower Bound	Upper Bound
Overall Agreement	0.049	0.008	5.878	<.001	0.033	0.066

Note that the agreement metrics reflect the agreement regarding the intrinsic and extrinsic explanation methods. Poor agreement in this context means that there is no consensus among participants on a preferred explanation method, i.e., they differ in their opinion between intrinsic and extrinsic explanation methods.

### Likert scale responses

**Fig 4:**
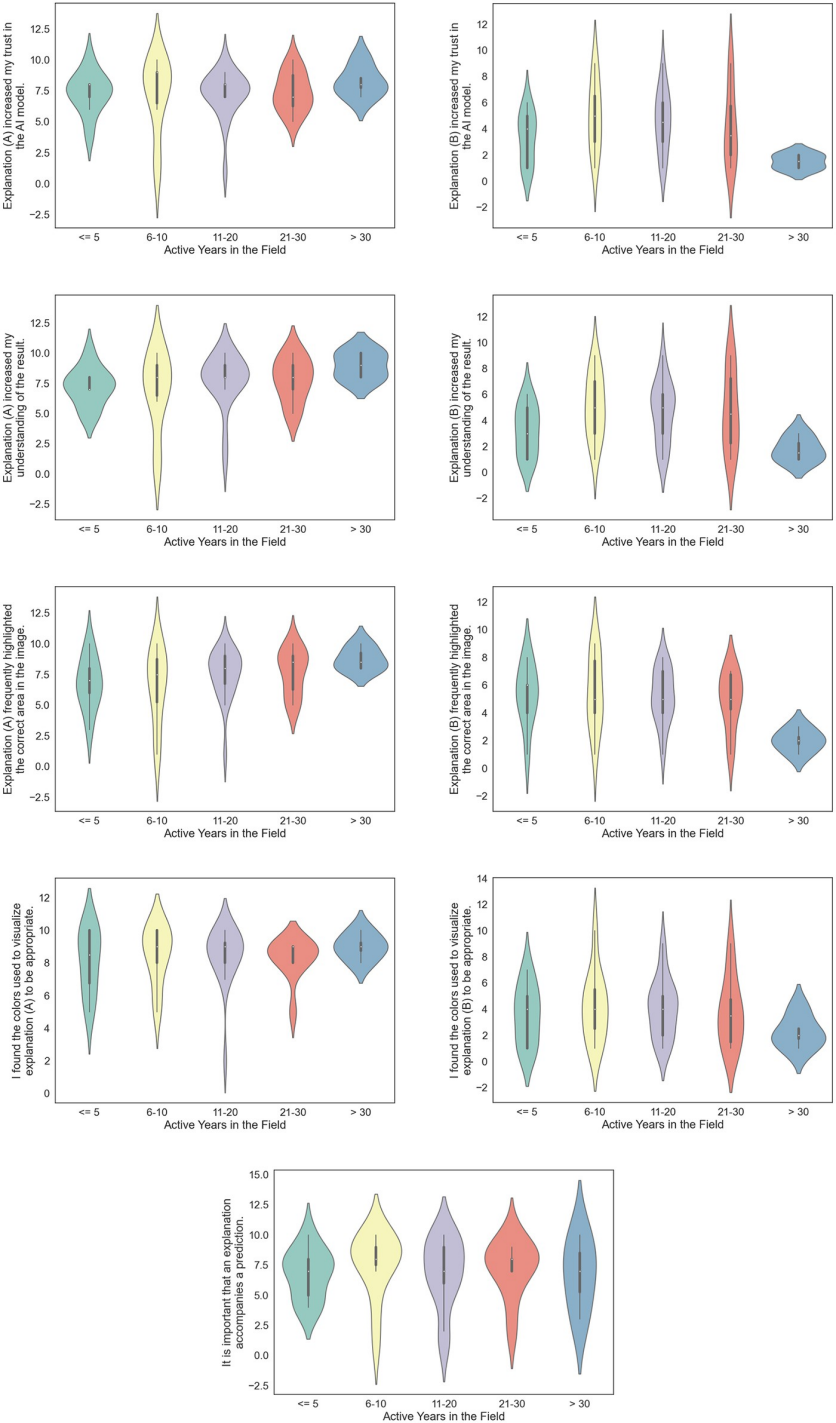
A collection of violin plots that presents an overview of the responses collected from the Likert questions. The answers are grouped by the number of years the person has been active in the medical field.

Fig 4 shows a collection of violin plots that showcase the answers collected from the Likert questions asked in the survey. Comparing the plots asking about increasing trust in the model for each respective method (Fig 4a and 4b), we see that there generally seems to be more agreement that the intrinsic method induces more trust in the underlying model across all experience groups. This pattern continues when comparing the plots for understanding (Fig 4c and 4d), spatial relevance (Fig 4e and 4f), and color choices (Fig 4g and 4h).

### Multiple-choice responses

**Fig 5:**
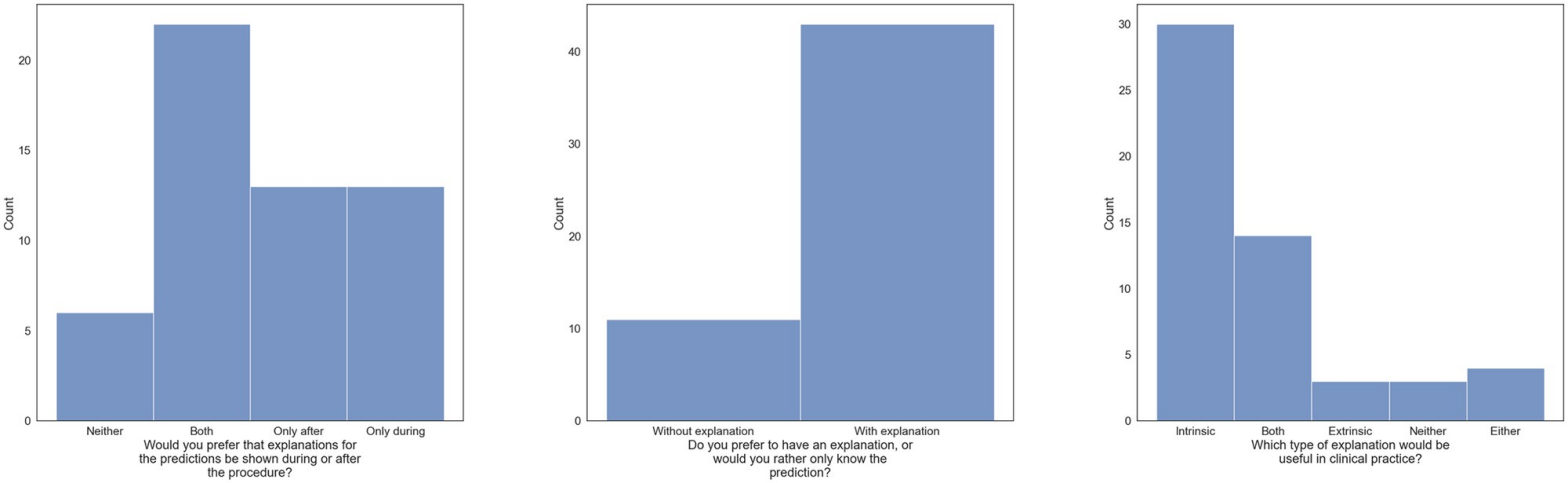
The responses collected from the multiple-choice questions asked in the survey. (a) Explanations during or after. (b) Prediction with or without explanation. (c) Most useful explanation in clinical practice.

According to the histogram plots in Fig 5a, most of the participants preferred to see the explanations of the model during and after the procedure, and the explanations were preferred to the absence of explanations; see Fig 5b. From Fig 5c, we see that 30 participants answered that the intrinsic explanation method would be useful in clinical practice, 14 participants wanted both explanation methods, while 3 wanted the extrinsic explanation method. 3 participants answered that none of the explanation methods would be useful and 4 of them answered that either one or the other method would be useful.

### Free-form responses

The survey included three questions with free-form responses, i.e., parts of the form where the participants could form their responses and elaborate freely. The three questions were

- Do you prefer to have an explanation, or would you rather only know the prediction?
- What do you think of explanation method A?
- What do you think of explanation method B?

and we provide a summary of the responses to each question below.

#### Do you prefer to have an explanation, or would you rather only know the prediction?

One participant stated regarding explainability as a “reasonable and popular expectation of AI systems”, but highlighted as a challenge that we humans when presented an explanation “make human interpretations/assumptions around what the explanation indicates about the underlying AI process [although] these interpretations may not actually be an accurate reflection of what is actually happening”, along with a reference to [25].

Another participant pointed out that “in clinical practice we do not have enough time to check the explanation during colonoscopy.”. A third contrasted clinical work with research, stating that they prefer no explanation during clinical work but receiving an explanation when doing research.

#### What do you think of explanation method A?

As indicated by the results of the Likert scale, most of the respondents prefer the intrinsic explanation method, which was reflected in their free-form responses. Participants who had positive opinions for Method A described it using terms such as “understandable”, “user friendly”, “helpful”, and “intuitive”.

We find that the following selection of the free-form answers represents the main reasons for which participants liked explanation method A: “easy to distinguish the red (interesting) areas from the other areas that are not as important.”, “Logical and a method that I have experienced with other examination modalities”, “I find it visually easier to understand and it pinpoints exactly what it is reacting to so it is easy to double-check the data.”, “It is simple and easy to understand which part of image should be focused on.”. Our interpretation of these and similar responses is that the saliency map produced by intrinsic explanation is visually intuitive and appealing and, therefore, possibly also used in other applications the participants may be familiar with.

On the other hand, the positive assessment of explanation Method A might depend strongly on the model prediction being correct. One participant stated that Method A is “Easy to understand, the red part is mostly in the same spot as the lesion.”, suggesting that the assessment would have been different had the model not identified the polyp. Another participant’s answer “It helped me identify important areas.” supports this notion.

Still, two participants wrote that “I think it is the best one to help you focus on the area that the AI system has identified as a suspected polyp.” and “The red color doesn’t show up where the polyp is (…) so for someone used to identify polyps in colonoscopy [this doesn’t indicate] that the program is able to really identify the polyp”, indicating that domain experts could use this explanation method to evaluate the machine learning model.

Among the responses giving method A a negative evaluation, one participant described the method as “sensitive but less specific” and another stated that it is “Sometimes (…) a bit intense and difficult to interpret”. One participant complained about the accuracy of method A, stating that “I prefer method A over B, but the poor accuracy of the method makes the explanation method (A) annoying rather than helpful.”. Finally, a participant stated that they “Did not get an explanation”, which we interpret as alluding to the fact that highlighting the information that goes into a decision is not sufficient to actually explain it.

#### What do you think of explanation method B?

As the responses to the Likert scale questions indicate, the study participants preferred the intrinsic explanation over the extrinsic explanation. Based on similar free-form answers from several participants, describing Method B as “complicated”, “confusing”, “hard to interpret”, “hard to understand”, “doesn’t feel natural”, and “harder to grasp visually”, we conclude that the extrinsic explanations functionality of highlighting in which direction each collection of image pixels drives the prediction is counterintuitive to domain experts not familiar with such a way to represent information. One participant also stated that “It is difficult to understand where to pay attention. There are several green spots in one image.”. Even though this indicated that the extrinsic explanation is not preferred by domain experts, it does not mean that the method does not provide value.

Before participating in the study, the participants were given an introduction to each explanation method, but it seems that a short brief is not enough to become comfortable with the visualizations that the extrinsic explanation produces, as supported by one participant’s statement “The image is messy. I understand the method as explained, but the method makes no sense to me.”, and another’s “(…) hard for my brain to wrap itself around the red/green ‘type of data in agreement or not’ paradigm used for this explanation”. Another stated that the method is “Harder to understand—however after a while you get a hang of it”, indicating that more time spent contemplating the method or studying several examples could have a strong positive effect on the evaluation of the method by medical doctors. This notion is supported by participants stating that method B “helps trust the system as it is based on the data” and “Makes more sense in terms of how data is trained”. The participants with positive sentiments towards explanation method B described it using terms such as “Interesting”, “intriguing” and “more specific”.

Furthermore, it seems that the choice of colormap, as well as super pixel size, could be adapted to better suit the end-users, as some participants stated that method B has “not the best colors”, is “confusing with the large amount of boxes not as pleasing to the eye”, and one suggests that “colors should be opposite. Red for disease, green for healthy.”. The latter response indicates that the particular participant had misunderstood the method, as the extrinsic method coloring indicates agreement with the model prediction; not the label—and consequently that method B is not sufficiently intuitive, as recently discussed. A participant also expressed concern about the suitability of this method for color-blind people. We have not taken this aspect into account in our study, but stress that, in general, any visualization method should abide by the principles of universal design, including color blindness accessibility.

## Discussion

In general, the answers to the free-form questions are in alignment with the responses to the Likert and multiple choice questions. Most doctors prefer intrinsic-based explanations as they are more easily aligned with their expectations in terms of spatial relevance and visual presentation. Participants found the intrinsic explanations to be more intuitive and user-friendly, and the visualizations more correctly aligned with their preconceived notions regarding what they expected the model to react to. Some specifically state that they prefer the intrinsic explanation as it more accurately highlights the lesion. The problem here is that the explanations are not there to detect subjects in an image but rather to explain why a specific prediction was made. If doctors expect explanations to always align with the object in question, explanations can hinder adoption and trust. Regarding extrinsic explanations, several physicians were confused by the visualizations and found them difficult to interpret, somewhat defeating the explanation in the first place. Some referenced the choice of colors and that the superpixels were not pleasing to look at. In contrast, there seems to be a different level of understanding in terms of AI knowledge among participants, some mentioning that they prefer the extrinsic explanation method due to it providing more information about how the model was trained. Perhaps the superficial aspects of the explanation could be improved by involving potential end-users in the development process to tailor the explanations to fit their use case and needs. By having a human-centered approach to generate explanations for AI systems, explanations may be regarded more useful to end users [26]. Regarding the explanations in general, the study participants preferred that the explanations be provided together with the model predictions (Fig 5), but what they considered the best explanation method varied between the participants. The human factor is important when developing model explanations [4]. What is regarded as a useful explanation by one person might not be so for another person. Consequently, subjective preferences might explain why the medical experts who participated in the study did not prefer the same model explanation.

### Limitations of the study

Although our study adds value to the field by evaluating common model explanations among medical experts, there are some limitations to consider. First, only two XAI methods were investigated: GradCAM and SHAP. Although a large number of methods have been proposed, it was infeasible to investigate all of them in this user survey. Still, the choice of which XAI methods to explore was not arbitrarily, but was based on the popularity of methods to explain deep neural networks in image analysis. We also wanted to compare extrinsic and instrinsic explanation methods, making GradCAM and SHAP natural choices. Although there is some uncertainty as to whether the feedback from these two types of explanation is representative of all salient-based explanation methods, we believe that the evaluation of frequently applied methods provides important insights into the current state of XAI in medicine. For future work, it would be relevant to look at other groups of explanations apart from saliency maps, including counterfactual and concept-based explanations.

Because our study was conducted in the field of GI diseases, we cannot guarantee that the results generalize to the medical field as a whole. Differences between specialties in medicine, such as data modalities, traditions, and accepted level of uncertainty in the analysis results, might affect what the medical professionals require from model explanations. Still, some preferences discovered in our study, for example, regarding the colors and superpixel layout of the explanations, are less dependent on the specific medical use case and will probably generalize to other medical domains. Furthermore, our findings indicate that giving a thorough introduction to AI and the XAI methods is necessary to ensure that medical experts can exploit the full potential of the model predictions and corresponding explanations. This would benefit healthcare personnel regardless of which diseases they work with, not only those who specialize in diseases in the GI tract. Furthermore, only ten image cases were shown to the study participants. This limitation comes from the time availability constraints of our study participants; a higher number of cases would lead to a lower number of participants. Although more showing more cases would be preferable, it was more important to have more participants. Finally, factors such as previous knowledge about AI among study participants, personal preferences, and how questions were framed can potentially affect the results. We designed the questions to be neutral and capture the relevant aspects of the explanations. Medical experts who are already interested in AI may be more motivated to participate in our study. However, free responses indicate large variations in how well participants understood the explanations they evaluated. Based on this, we regard the recruited participants to be diverse and well represented regarding AI literacy.

As previously stated, how well an explanation is perceived is subjective. The results of the survey will, consequently, be affected by the personal preference or if the medical experts have had previous experiences with AI. In our view, this is not a limitation, but rather a strength. Being able to capture subjective preferences in a group of experts enables us to tailor the model explanations to their expectations in a way that would not be possible by using objective evaluation metrics. Here, it is important to note that our research emphasizes qualitative evaluation, prioritizing depth of analysis over statistical power. However, we acknowledge that putting too much emphasis on preferences from only one or a couple of users might lead to a design that is not well received by everyone. Since our study included more than 50 medical experts from the GI field, we believe the general trends in preferences will be useful to explain future AI systems for the detection of GI diseases.

Despite the above-mentioned limitations, the study makes a significant contribution to the computer science and medical communities by providing initial insights into medical professionals’ preferences for AI explanation methods in GI imaging. This is one of the most advanced medical fields in the context of AI, highlighting the importance of user-centric design and the need for a larger study that explores how AI explanation methods can be used in a clinical setting.

## Conclusion

This paper presents a study comparing intrinsic and extrinsic explanation methods from the perspective of medical doctors. The study was carried out using a GI use case that involved explanations of a machine learning model used to predict polyps in images. Study participants were gathered from different parts of the world to complete a survey consisting of model predictions accompanied by two explanation methods for ten different medical cases. Our results show that intrinsic explanations are preferred. However, the free-form responses in our survey strongly suggest that the underlying reason for the doctors’ preference for this method may be more superficial than actually understanding what information the different explanations convey. This suggests that a certain level of training or practice is required for physicians to fully exploit the usefulness of ML model explanations, although we might naïvely expect that all image-based explanations are sufficiently intuitive to be useful without prior training. We highlight that any form of explanation targeted at non-technical end-users, such as doctors, must be developed with the end-user in mind, ideally also involving the end-user. This includes following the principles of universal design to accommodate specific needs. In conclusion, medical professionals recognize the usefulness of visual explanations for deep learning-based computer vision models, but limited understanding and reasoning behind an explanation may lead to unwarranted judgments based on wrong principles. Using the insights acquired in this study, we are better equipped to design medical AI systems that are also well explained to healthcare personnel. Hopefully, this will facilitate the implementation of such systems in the clinic and contribute to better treatment and follow-up of patients in the GI domain and beyond.

## Supporting information

S1 Data The unprocessed responses from participants, which include answers to the survey and the voting on intrinsic versus extrinsic explanation methods. (CSV)
